# Posterior cerebral fossa medulloepithelioma: report of a case

**DOI:** 10.1186/s12907-017-0064-x

**Published:** 2017-11-21

**Authors:** Nezha Oumghar, Fatima Ezzahra Hazmiri, Abdelhamid El Omrani, Hanane Rais, Mouna Khouchani

**Affiliations:** 1 Radiotherapy department – Oncology and hematology center, Mohammed VI University Hospital, Marrakech, Morocco; 2 Pathology department – Arrazi Hospital, Mohammed VI University Hospital, Marrakech, Morocco

**Keywords:** Medulloepithelioma, Primitive neuroectodermal tumor, Infratentorial, Radiotherapy

## Abstract

**Background:**

Medulloepithelioma is a rare primitive neuroectodermal tumor of the central nervous system, usually developing in childhood. Due to its rarity, the optimal management is still unknown. The prognosis is poor, especially when resection is incomplete. Adjuvant radiochemotherapy is often indicated.

**Case presentation:**

We report a rare case of infratentorial medulloepithelioma in a 3 year old girl. She presented symptoms of increased intracranial pressure. On examination, she had coordination problems, ptosis and exotropia of the right eye. Magnetic resonance imaging demonstrated a large cerebellar vermix tumor. Immuno-histochemistry revealed a diffuse positivity for Vimentin and focal positivity for the epithelial membrane antigen, but Glial Fibrillary Acidic Protein and Synaptophysin were negative, the MIB-1 antibody was very high. She received postoperative craniospinal irradiation and died 7 months later.

**Conclusion:**

We describe the features (epidemiological, clinical, histological, immunohistochemical and therapeutic outcomes) of our case and confront it to literature data.

## Background

Medulloepithelioma of the central Nervous System (CNS) is an uncommon primitive neuroectodermal tumor (PNET), characterized by a highly malignant behavior [1]. It is a rare tumor usually located in the supratentorial area [2], with the peak incidence between 6 months to 5 years of age. We report a case of infratentorial medulloepithelioma in a 3-year-old girl.

## Case presentation

**Fig. 1:**
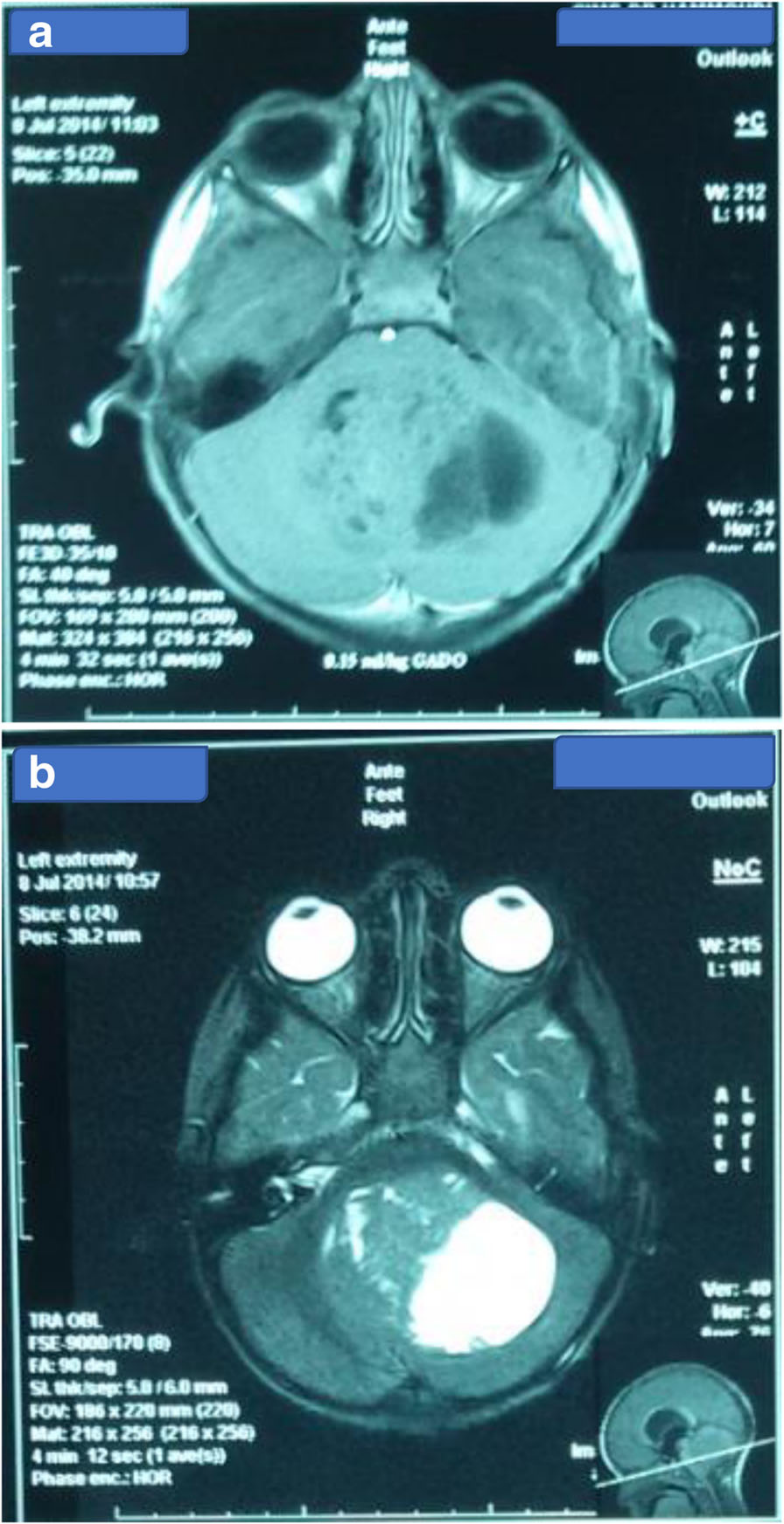
The axial T1 (**a**), T2 W/FLAIR (**b**) image shows cerebellar vermis lesion that was hypointense on T1WI and heterogeneous on T2WI, vith perilesional edema, and triventricular hydrocephalus

**Fig. 2:**
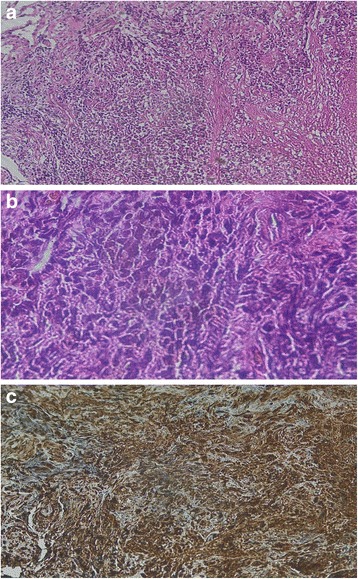
Histological and Immunohistochemical findings. **a:** Malignant tumor proliferation with round cells HE x10 **b:** Tubular structures HEx20 **c:** Intense and diffuse membrane expression of vimentin HEx10

A 3 year-old girl was admitted to the emergency department with intracranial hypertension picture without fever of 30 days duration. On examination, she had coordination problems, ptosis and exotropia of the right eye. Her vitals were stable. Brain magnetic resonance imaging (MRI) showed a large cerebellar vermis lesion compressing the 4th ventricle with surrounding edema and triventricular hydrocephalus, the mass demonstrated a heterogeneous low signal on T2-weighted (Fig. 1). A subtotal resection and ventriculoperitoneal shunt hydrocephalus was performed. Histology characteristically shows a Highly cellular embryonal tumor composed of small size cells with little perinuclear cytoplasm, and Hyperchromatic nuclei with granular chromatin. Mitotic figures are abundant. it is also characterized by the presence of islets of hyaline cartilage and tumoral necrosis. Immunohistochemistry revealed diffuse positivity for Vimentin, while expression of synaptophysin was absent. It showed focal positivity for the Epithelial Membrane Antigen (EMA), for AC anti-EMA and for AC anti- PS100, while the Glial Fibrillary Acidic Protein (GFAP) was negative. MIB-1 labeling was very high, exceeding 50%. The diagnosis of medulloepithelioma was retained (Fig. 2). The evolution has been marked by the deterioration of neurological status, she had corticicosteroide bolus without improvement of symptoms, she received a craniospinal irradiation: The patient underwent a planning CT in dorsal decubitus position under sedation, The treatment was delivered via 2 lateral fields to the head and 2 direct posterior fields to upper and lower spine, The junctions between the 3 fields moved caudally every week, 36Gy in 2-Gy fractions were delivered over 4 weeks to the craniospinal axis, with a boost to the posterior fossa, Radiotherapy was well tolerated with a spectacular improvement of symptoms, during radiotherapy. Neurological condition deteriorated 3 months after the end of radiotherapy and survival was 6 months.

## Discussion

The medulloepithelioma of the central nervous system is a rare tumor representing 1.5% of primary tumors of the CNS [3], with a peak incidence between 6 months and 5 years of age [4], few cases in adolescents are reported in the literature. No specific sex predilection [2, 5].

Medulloepitheliomas are mostly supratentorial lesions commonly found in the periventricular location [6]. They are mostly involving in order of frequency: temporal, parietal, occipital and frontal lobes. Other locations have been reported in the literature such as in infratentorial: at the level of the brainstem [4], or cerebellar pontine angle [7]. The intraorbital location is quite special because of the origin remains unexplained and good prognosis after enucleation of the eye [4].

The clinical picture is nonspecific and is based on the seat of the lesion. Most often, it is a syndrome of intracranial hypertension with or without a neurological deficit. The mean duration of symptoms was 6 weeks [8].

Appearances on MRI are very variable, they are typically iso to hypointense with grey matter on T1 weighted imaging and iso to hypointense on T2 weighted imaging, 95% of tumours will show contrast enhancement, particularly of the solid elements [9]. Pre-operative imaging with MRI is important in order to assess CSF dissemination of tumour.

The diagnosis of medulloepithelioma can be confusing with other primitive neuroectodermal tumors (PNET), such as medulloblastoma, neuroblast, the ependymoblastoma, teratoma or some glial tumors [10]. The predominant structure resembles that of the epithelium of the primitive neural tube. Besides the beaches of neuroepithelial progenitor cell, papillary and / or tubular formations and neuroepithelial cells arranged in a pseudo-epithelium laminate are found [10, 11]. The tumor cells are relatively large, cylindrical or polygonal and have scant eosinophilic cytoplasm. The nuclei are oval or rounded vary in size with a granular chromatin with many nucleoli [12]. Figures mitotic often are many particularly at the level of cells adjacent to the luminal surface [13]. The latter is covered by a PAS positive material simulating internal limiting membrane [12]. The outer surface of these tubes is limited by a basement membrane or outer limiting membrane resting on a loose connective tissue stroma. Neuronal maturation areas, glial, ependymal, or even mesenchymal are reported. Histochemical techniques and immunohistochemical provided an additional argument in differentiation potential of this neoplasm. Thus, the ependymal and astroglial elements have positive immunoreactivity to GFAP and PS100, and neural elements have a positive immunoreactivity for synaptophysin. An important fact is the rather frequent positive immunoreactivity to vimentin [4], which is the first protein filamentary intermediate expressed at an early stage of development of the neural tube mammals. Finally, the beaches of necrosis and hemorrhage can be found. This tumor has an aggressive local infiltrative extension and a tendency to spread along drainage ways CSF [14]. Some authors have reported metastases extra Nevrax.

The prognosis of these tumors is generally poor due to their relative radioresistance and their inexorable tendency to recurrence and spread to intra-and extra-axial [15]. The mean survival is estimated by the literature to 5 months [4]. However very long survival exceeding 13 years have been reported, In a recent review of the literature [2, 15], 44 cases of medulloepithelioma were published by authors from different interest poles [16], according to this study, the best treatment of these lesions appears is the most complete surgical excision associated with extensive entire neuraxis radiotherapy, the dose prescription for patients with localised PNETs should be 35 Gy in 21 daily fractions, followed by a second radiotherapy phase of 20 Gy in 12 fractions to the posterior fossa or otherwise to the primary tumour (if biopsied only) or any postoperative residual tumour. Adjuvant chemotherapy can have a beneficial effect for some authors but the systematic indication remains controversial. The best results obtained in this type of tumor may be related to some factors predictive of good prognosis: Supratentorial location, gross total resection, absence of cerebrospinal fluid (CSF) dissemination, and postoperative aggressive chemoradiotherapy [2, 14, 17, 18].

Table 1 summarizes the treatments received and the survival in cases of medulloepithelioma of the posterior cerebral fossa published in the literature [4, 7, 10, 12, 19–22].

**Table 1:** Characteristics of the published cases of posterior cerebral fossa medulloepithelioma

Case	Age	Dissemination	Surgery	RT	CT	Survival
Best (1974) [19]	2 years	No	Biopsy	No	No	2 weeks
Pollak et Friede (1977) [12]	15 months	NR	PR	Yes	No	11 months
Bonnin et al. (1984) [20]	15 months	LCR	PR	No	No	4 months
Molloy et al. (1996) [4]	15 months	No	TR	Yes	Yes	> 13 years
Khoddami et Becker (1997) [21]	30 months	NR	PR	Yes	No	11 months
Scharma et al. (1998) [22]	9 months	No	TR	Yes	No	NR
Vincent et al. (2002) [10]	3 months	NR	TR	No	No	> 7 years
Syal et al. [7]	5 years	LCR	TR	Yes	Yes	< 1 month
Our patient (2014)	3 years	No	PR	Yes	No	6 months

*RT* radiotherapy, *CT* chemotherapy, *PR* partial resection, *TR* total resection, *LCR* cerebrospinal fluid, *NR* non reported

Necessity to look for alternative molecular markers and directed treatment similar to INI-1 in atypical teratoid rhabdoid tumors is emerging. In recent times, hTERT oncogene amplification has been demonstrated in a proportion of embryonal tumors, particularly medulloblastomas, as well as, medulloepitheliomas [23], a correlation with survival has been suggested and whether this might emerge as a molecular marker in future remains to be seen.

## Conclusion

The medulloepithelioma is the most undifferentiated and malignant of PNET. It affects young children and can sit anywhere in the CNS, usually in periventricular. Clinical and imaging are not specific. Only histology with immunohistochemistry confirms the diagnosis by showing the typical appearance, with often positive immunoreactivity to vimentin. The prognosis may be better if we can make a complete surgical resection and radiotherapy.

### Informed consent

Written informed consent was obtained from the parents of patient for publication of this case report and any accompanying images. A copy of the written consent is available for review by the Editor-in-Chief of this journal.

### Patient perspective

Inappropriate.

## Abbreviations

CNS: Central Nervous System
CSF: Cerebrospinal fluid
EMA: Epithelial Membrane Antigen
GFAP: Glial Fibrillary Acidic Protein
PNET: Primitive neuroectodermal tumor
